# Substituting random forest for multiple linear regression improves binding affinity prediction of scoring functions: Cyscore as a case study

**DOI:** 10.1186/1471-2105-15-291

**Published:** 2014-08-27

**Authors:** Hongjian Li, Kwong-Sak Leung, Man-Hon Wong, Pedro J Ballester

**Affiliations:** Department of Computer Science and Engineering, Chinese University of Hong Kong, Hong Kong, China; European Bioinformatics Institute, Wellcome Trust Genome Campus, Hinxton, Cambridge, UK; Cancer Research Center of Marseille (Inserm U1068, UM105, IPC), 27 Boulevard Lei Roure, 13009 Marseille, France

**Keywords:** Molecular docking, Binding affinity, Drug discovery, Machine learning

## Abstract

**Background:**

State-of-the-art protein-ligand docking methods are generally limited by the traditionally low accuracy of their scoring functions, which are used to predict binding affinity and thus vital for discriminating between active and inactive compounds. Despite intensive research over the years, classical scoring functions have reached a plateau in their predictive performance. These assume a predetermined additive functional form for some sophisticated numerical features, and use standard multivariate linear regression (MLR) on experimental data to derive the coefficients.

**Results:**

In this study we show that such a simple functional form is detrimental for the prediction performance of a scoring function, and replacing linear regression by machine learning techniques like random forest (RF) can improve prediction performance. We investigate the conditions of applying RF under various contexts and find that given sufficient training samples RF manages to comprehensively capture the non-linearity between structural features and measured binding affinities. Incorporating more structural features and training with more samples can both boost RF performance. In addition, we analyze the importance of structural features to binding affinity prediction using the RF variable importance tool. Lastly, we use Cyscore, a top performing empirical scoring function, as a baseline for comparison study.

**Conclusions:**

Machine-learning scoring functions are fundamentally different from classical scoring functions because the former circumvents the fixed functional form relating structural features with binding affinities. RF, but not MLR, can effectively exploit more structural features and more training samples, leading to higher prediction performance. The future availability of more X-ray crystal structures will further widen the performance gap between RF-based and MLR-based scoring functions. This further stresses the importance of substituting RF for MLR in scoring function development.

**Electronic supplementary material:**

The online version of this article (doi:10.1186/1471-2105-15-291) contains supplementary material, which is available to authorized users.

## Background

Protein-ligand docking is a computational tool that predicts how a ligand binds to a target protein and their binding affinity. Hence docking is useful in elaborating intermolecular interactions and enhancing the potency and selectivity of binding in subsequent phases of computer-aided drug design. Docking has a wide variety of pragmatic and successful applications in structure-basedvirtual screening [1], drug repurposing [2], lead compound optimization [3], protein cavity identification [4], and protein function prediction [5].

Docking consists of two major operations: predicting the position, orientation and conformation of a ligand when docked to the protein’s binding pocket, and predicting their binding strength. The former operation is known as pose generation, and the latter is known as scoring. State-of-the-art docking methods, such as AutoDock Vina [6] and idock [7], work reasonably well at pose generation with a redocking success rate of over 50% [8] on the benchmarks of both PDBbind v2012 and v2011 [9, 10] and the CSAR NRC HiQ Set 24 Sept 2010 [11, 12]. However, the single most critical limitation of docking is the traditionally low accuracy of the scoring functions.

Classical scoring functions are defined by the assumption of a fixed functional form for the relationship between the numerical features that characterize the protein-ligand complex and its predicted binding affinity. This functional form is composed of the energetic contributions of various intermolecular interactions, and is often additive. The overall binding affinity is calculated as a weighted sum of several physically meaningful terms, while their coefficients are typically derived from standard multivariate linear regression (MLR) on experimental data.

Cyscore [13], a recently published empirical scoring function, assumes that the overall protein-ligand binding free energy can be decomposed into four terms: hydrophobic free energy, van der Waals interaction energy, hydrogen bond interaction energy and ligand’s conformational entropy. Cyscore focuses on improving the prediction of hydrophobic free energy by using a novel curvature-dependent surface-area model, which was claimed to be able to distinguish convex, planar and concave surface in hydrophobic free energy calculation.

A recent study on a congeneric series of thrombin inhibitors concludes that free energy contributions to ligand binding at the molecular level are non-additive [14], therefore the modelling assumption of additivity models is error prone. Recent years have seen a growing number of new developments of machine-learning scoring functions, with RF-Score [15] being the first that introduced a large improvement over classical approaches. RF-Score, as its name suggests, uses Random Forest (RF) [16] to implicitly learn the functional form in an entirely data-driven manner, and thus circumvents the modelling assumption imposed by previous scoring functions. RF-Score was shown to significantly outperform 16 classical scoring functions when evaluated on the common PDBbind v2007 benchmark [15]. Despite being a recent development, RF-Score has already been successfully used to discover a large number of innovative binders against antibacterial DHQase2 targets [17]. For the purpose of prospective virtual screening, RF-Score-v3 has now been incorporated into istar [8], our large-scale docking service available at http://istar.cse.cuhk.edu.hk/idock. A number of subsequent machine-learning scoring functions, including NNScore [18], SVR-KB and SVR-EP [19], CScore [20], B2Bscore [21], SFCscoreRF [22], and ID-Score [23], have also shown large improvements over classical approaches.

In this study we compare the prediction performance of two regression models MLR and RF (to be exact, random forest regression rather than classification), and investigate their application conditions and interpretability under various contexts. The Methods section introduces MLR and RF, three sets of features, three benchmarks, two kinds of cross validations, and four performance metrics. The Results and discussion section analyzes the prediction performance of MLR and RF on the three benchmarks and discusses the conditions of applying MLR and RF. The Conclusions section emphasizes the importance of abundance of features and samples for training RF.

## Methods

### Multiple linear regression (MLR) with Cyscore features

Cyscore is an empirical scoring function in an additive functional form of four energetic terms, which are hydrophobic free energy *ΔG*_*hydrophobic*_, van der Waals interaction energy *ΔG*_*vdw*_, hydrogen bond interaction energy *ΔG*_*hbond*_ and ligand’s conformational entropy *ΔG*_*entropy*_ (Eq. 1). Their coefficients *k*_*h*_, *k*_*v*_, *k*_*b*_ and *k*_*e*_ and the intercept *C* were obtained by MLR on 247 high-quality complexes carefully selected from PDBbind v2012 refined set. The intercept value was not reported in the original publication, but was included in this study as usual [24] in order to make a quick estimation of absolute binding affinity value, which is the ultimate goal in some real-world applications.
1

We use MLR::Cyscore to denote the scoring function built with MLR and the 4 features from Cyscore. It is noteworthy that Cyscore is a pure MLR model, unlike AutoDock Vina [6] which is a quasi MLR model because the number of rotatable bonds *N*_*rot*_ is in the denominator so as to penalize ligand flexibility (see [8] for the exact equation) and therefore MLR::Vina would require an additional grid search for the weight of the *N*_*rot*_ parameter. So this study allows a more direct comparison between MLR and RF.

### Random forest (RF) with Cyscore, AutoDock Vina and RF-score features

A RF [16] is a consensus of a large number of different decision trees generated from random bootstrap sampling of the same training data. During tree construction, at each inner node RF chooses the best splitting feature that results in the highest purity gain from a normally small number (mtry) of randomly selected features rather than utilizing all input features. In regression problems, the final output is calculated as the arithmetic mean of all individual tree predictions in the RF. Further details on RF construction can be found in [8, 15].

In this study, multiple RFs of the default number of 500 trees were built using values of the mtry control parameter from one to the total number of input features. The selected RF was the one resulting in the lowest root mean square error (RMSE) on the Out-of-Bag (OOB) samples of the training set. Only one single random seed was used for training because seed is not a significant impact factor of the prediction performance, and using fewer seeds has the additional advantage of leading to computationally faster training process.

In our experiments we aimed at analyzing how RF responds to varying numbers of features and hence we selected three sets of features: Cyscore [13], AutoDock Vina [6] and RF-Score [15]. Cyscore comprises four numerical features: *ΔG*_*hydrophobic*_, *ΔG*_*vdw*_, *ΔG*_*hbond*_ and *ΔG*_*entropy*_. AutoDock Vina comprises six numerical features: *Gauss*_1_, *Gauss*_2_, *Repulsion*, *Hydrophobic*, *HBonding* and *N*_*rot*_. RF-Score comprises 36 features, defined as the occurrence count of intermolecular contacts between two elemental atom types. Four atom types for proteins (C, N, O, S) and nine for ligands (C, N, O, S, P, F, Cl, Br, I) were selected so as to generate dense features while considering all the heavy atom types commonly observed in protein-ligand complexes. Table 1 summarizes the three combinations of these feature sets used to train RF models. Altogether four models (MLR::Cyscore, RF::Cyscore, RF::CyscoreVina and RF::CyscoreVinaElem) were evaluated in this study.

**Table 1:** **The three combinations of three different sets of features used to train RF models in this study**

Model	Features
RF::Cyscore	4 Cyscore features
RF::CyscoreVina	4 Cyscore features + 6 AutoDock Vina features
RF::CyscoreVinaElem	4 Cyscore features + 6 AutoDock Vina features +
	36 RF-Score features

### PDBbind v2007 and v2012 benchmarks

The PDBbind [9, 10] benchmark is arguably the most widely used for binding affinity prediction. It contains an especially diverse collection of experimentally resolved protein-ligand complexes, assembled through a systematic mining of the yearly releases of the entire PDB [25, 26]. For each complex, the experimentally measured binding affinity, either dissociation constant Kd or inhibition constant Ki, was manually collected from its primary literature reference. The complexes with a resolution of ≤2.5Å and with the ligand comprising merely nine common heavy atom types (C, N, O, F, P, S, Cl, Br, I) were filtered to constitute the refined set. These complexes were then clustered by protein sequence similarity with a cutoff of 90%, and for each of the resulting clusters with at least five complexes, the three complexes with the highest, median and lowest binding affinity were selected to constitute the core set. Because of the structural diversity of the core set, it is a common practice to use the core set as a test set and the remaining complexes in the refined set as a training set.

On one hand, Cyscore was tested on two independent sets: PDBbind v2007 core set (N = 195) and PDBbind v2012 core set (N = 201), whose experimental binding affinities span 12.56 and 9.85 pKd units, respectively. On the other hand, Cyscore was trained on a special set of 247 complexes carefully selected from the PDBbind v2012 refined set using certain criteria [13] (e.g. structural resolution < 1.8Å, binding affinity spans 1 to 11 kcal/mol, protein sequence similarity and ligand chemical composition are different from the test set), ensuring that the training complexes are of high quality and do not overlap with any of the two test sets. In this study we used exactly the same training set and the same test sets in order to make a fair comparison to Cyscore.

Furthermore, considering the fact that 16 classical scoring functions have already been evaluated [24] on PDBbind v2007 core set and the top performing of them (e.g. X-Score) were trained on the remaining 1105 complexes in PDBbind v2007 refined set, we also used these 1105 complexes as another training set to permit a direct comparison. Using predefined training and test sets, where other scoring functions had previously been trained and tested, has the advantage of reducing the risk of using a benchmark complementary to one particular scoring function.

Likewise for the PDBbind v2012 benchmark, we used an additional training set comprising the complexes in PDBbind v2012 refined set excluding those in PDBbind v2012 core set. This led to a total of 2696 complexes. By construction, this training set does not overlap with the test set.

### PDBbind v2013 round-robin benchmark

We propose a new benchmark to investigate how prediction performance of the four models changes in cross validation and with varying numbers of training samples. We used PDBbind v2013 refined set (N = 2959), which is the latest version and constitutes the most comprehensive and publicly available structural dataset suitable for training scoring functions.

We used 5-fold cross validation, as was used by the recently published empirical scoring function ID-Score [23], to reduce overfitting and thus generalization errors. The entire PDBbind v2013 refined set (N = 2959) was divided into five equal partitions using uniform sampling on a round-robin basis: the entire 2959 complexes were first sorted in the ascending order of their measured binding affinity, and the complexes with the 1st, 6th, 11th, etc. lowest binding affinity belonged to the first partition, the complexes with the 2nd, 7th, 12th, etc. lowest binding affinity belonged to the second partition, and so on. This partitioning method, though not completely random, has two advantages: on one hand, each partition is guaranteed to span the largest range of binding affinities and incorporates the largest structural diversity of different protein families; on the other hand, each partition is composed of a deterministic list of complexes, permitting reproducibility and comparisons in future studies. Table 2 summarizes the statistics of the five partitions. The PDB IDs and measured binding affinities of the complexes in the five partitions are available in the Additional file 1.

**Table 2:** **The statistics of the five partitions of PDBbind v2013 refined set (N = 2959)**

#	Complexes	Lowest pKd	Highest pKd
1	592	2.00	11.74
2	592	2.00	11.80
3	592	2.00	11.85
4	592	2.00	11.92
5	591	2.05	11.72

We then used the partition on which the best performance was obtained (It turned out to be partition 2 (N = 592). See the Results and discussion section.) as the test set in PDBbind v2013 round-robin benchmark, and used the remaining four partitions (1, 3, 4, 5) to construct four training sets of incremental sizes: the first training set comprises partition 1 (N = 592), the second training set comprises partitions 1 and 3 (N = 1184), the third training set comprises partitions 1, 3 and 4 (N = 1776), and the fourth training set comprises partitions 1, 3, 4 and 5 (N = 2367). Therefore this new benchmark provides a way to study how prediction performance varies with training set size. Moreover, its test set has a significantly larger number of complexes (N = 592) compared to PDBbind v2007 (N = 195) and v2012 (N = 201) benchmarks, making this new benchmark not being a redundant duplication of the previous two benchmarks. Table 3 summarizes the numbers of test and training samples for the three benchmarks.

**Table 3:** **The numbers of test samples and training samples for the PDBbind v2007, v2012 and v2013 benchmarks used in this study**

Benchmark	Test samples	Training samples
v2007	195	247, 1105
v2012	201	247, 2696
v2013	592	592, 1184, 1776, 2367

### Leave-cluster-out cross validation (LCOCV)

Leave-cluster-out cross validation (LCOCV) [27], in contrast to standard cross validation, divides the complete set of complexes into protein families instead of random subsets. Each protein family, or each cluster, is typically determined by 90% protein sequence identity. Protein families with at least ten complexes are treated as individual clusters, labeled as A to W. Protein families with four to nine complexes are combined into cluster X. Protein families with two to three complexes are combined into cluster Y. Singletons are combined into cluster Z. Each cluster is iteratively left out of the training set and used to evaluate the predictive performance of the scoring function. The performance on each cluster can be inspected individually, and the overall performance can be estimated by averaging over all clusters.

So far LCOCV has been applied to the assessment of six scoring functions, which are RF-Score [20, 21, 27], ddPLAT+MOE [28], CScore [20], B2Bscore [21], SFCscoreRF [22] and the work of Ross et al. [29].

For the purpose of comparison to other scoring functions, PDBbind v2009 refined set (N = 1741) was used in this study to perform LCOCV. The 1xr8 entry in cluster X was discarded because its ligand is far away from its protein, thereby leaving 1740 complexes. The PDB IDs and measured binding affinities of the complexes in the 23 protein families (A to W) and the 3 multi-family clusters (X to Z) are available in the Additional file 2.

### Performance metrics

Prediction performance was quantified through standard deviation SD in linear correlation, Pearson correlation coefficient Rp and Spearman correlation coefficient Rs between the measured and predicted binding affinities of the test set. These metrics are commonly used in the community [24], and the SD metric is essentially the residual standard error (RSE) metric used in some other studies [19]. The above three metrics are invariant under linear transformations (e.g. changing the intercept or coefficient values in Eq. 1 affects none of these metrics), so they are mainly for comparative purpose. In some applications, however, the ultimate goal of scoring functions is to report an absolute binding affinity value as close to the measured value as possible. Hence we use a more realistic metric, the root mean square error RMSE between measured and predicted binding affinities without a linear correlation. Lower values in RMSE and SD and higher values in Rp and Rs indicate better prediction performance.

Mathematically, equations 2, 3, 4 and 5 show the expressions of the four metrics. Given a scoring function *f* and the features  describing the *n*th complex out of *N* complexes in the test set,  is the predicted binding affinity,  are the fitted values from the linear model between {*y*^(*n*)^} and {*p*^(*n*)^} on the test set, whereas  and  are the rankings of {*y*^(*n*)^} and {*p*^(*n*)^}, respectively.
2345

## Results and discussion

**Figure 1:**
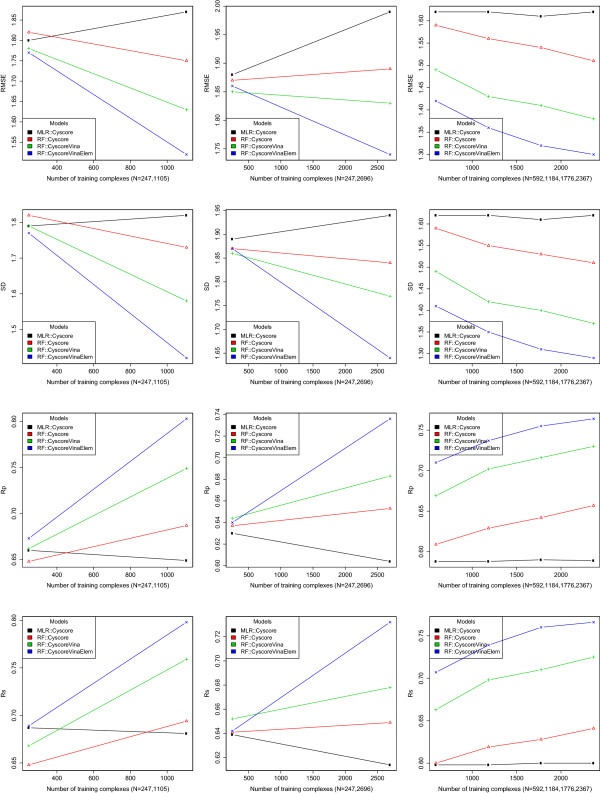
**Prediction performance of MLR::Cyscore, RF::Cyscore, RF::CyscoreVina and RF::CyscoreVinaElem trained with varying numbers of samples.** First row: root mean square error RMSE. Second row: standard deviation SD in linear correlation. Third row: Pearson correlation coefficient Rp. Fourth row: Spearman correlation coefficient Rs. Left column: PDBbind v2007 benchmark (N = 195). Center column: PDBbind v2012 benchmark (N = 201). Right column: PDBbind v2013 round-robin benchmark (N = 592).

Figure 1 plots the prediction performance of MLR::Cyscore, RF::Cyscore, RF::CyscoreVina and RF::CyscoreVinaElem using different numbers of training samples on PDBbind v2007 benchmark (N = 195), PDBbind v2012 benchmark (N = 201) and PDBbind v2013 round-robin benchmark (N = 592). The raw values are available in the Additional file 3.

### MLR::Cyscore performance does not increase with more training samples

On both PDBbind v2007 and v2012 benchmarks, MLR::Cyscore performed best when it was trained on the 247 carefully selected complexes used by Cyscore. Its performance dropped when more complexes were used for training. On PDBbind v2013 round-robin benchmark, MLR::Cyscore performance stayed flat regardless of training set sizes.

These results show that MLR::Cyscore is unable to exploit large sizes of structural data given only a small set of sophisticated features. Feeding more training samples to MLR::Cyscore actually increases the difficulty in regressing the coefficients well. Generally it would be a good idea to select the training complexes that provide the best performance on a test set, as was the case of Cyscore. However, in real applications the binding affinities of the test set are not known and unfortunately selection of training complexes is not performed blindly (i.e. without measuring performance on test set).

### RF performance increases with more structural features and training samples

On all the three benchmarks, given the same set of features, the RF models trained with more samples resulted in higher prediction accuracy. Similarly, given the same training samples, the RF models trained with more features resulted in higher prediction accuracy.

These results suggest that RF is capable of effectively exploiting a comprehensive set of structural features and training samples. Generally the more training samples, the more knowledge for RF to learn so as to capture the non-linearity of the structural data. Likewise, the more appropriate features, the higher probability of choosing the best splitting feature that can result in a high purity gain at non-leaf nodes during RF construction, and hence the higher chance of boosted RF performance.

### RF models perform consistently well in cross validation

Table 4 shows the results of 5-fold cross validation for all the four models. The best performance was obtained on partition 2. In terms of average performance, the relative performance ranking is consistent, where RF::CyscoreVinaElem (RMSE = 1.35, SD = 1.35, Rp = 0.738, Rs = 0.738) is better than RF::CyscoreVina (RMSE = 1.44, SD = 1.44, Rp = 0.693, Rs = 0.690), which is better than RF::Cyscore (RMSE = 1.59, SD = 1.59, Rp = 0.603, Rs = 0.587), which is better than MLR::Cyscore (RMSE = 1.66, SD = 1.66, Rp = 0.556, Rs = 0.559).

**Table 4:** **Cross validation results of the four models on the five partitions of PDBbind v2013 refined set (N = 2959) in terms of root mean square error RMSE, standard deviation SD in linear correlation, Pearson correlation coefficient Rp and Spearman correlation coefficient Rs**

				MLR::Cyscore				RF::Cyscore				RF::CyscoreVina				RF::CyscoreVinaElem	
#	N	RMSE	SD	Rp	Rs	RMSE	SD	Rp	Rs	RMSE	SD	Rp	Rs	RMSE	SD	Rp	Rs
1	592	1.66	1.66	0.560	0.555	1.60	1.60	0.601	0.588	1.41	1.41	0.708	0.709	1.33	1.33	0.748	0.746
2	592	1.62	1.62	0.589	0.600	1.51	1.51	0.657	0.641	1.38	1.37	0.730	0.725	1.30	1.29	0.764	0.766
3	592	1.69	1.70	0.531	0.529	1.66	1.66	0.561	0.545	1.49	1.49	0.668	0.665	1.41	1.41	0.711	0.709
4	592	1.68	1.68	0.542	0.557	1.63	1.63	0.580	0.576	1.51	1.51	0.657	0.661	1.41	1.41	0.711	0.722
5	591	1.65	1.65	0.559	0.553	1.57	1.57	0.615	0.586	1.42	1.42	0.701	0.692	1.30	1.30	0.758	0.749
avg		1.66	1.66	0.556	0.559	1.59	1.59	0.603	0.587	1.44	1.44	0.693	0.690	1.35	1.35	0.738	0.738

### Leave-cluster-out cross validation leads to unrealistically low performance

Table 5 shows the results of leave-cluster-out cross validation (LCOCV) for all the four models. Not unexpectedly, the observed performance is very heterogeneous across the different protein families. These results indeed agree with the LCOCV results of six other scoring functions from previous studies [20–22, 27–29]. By analyzing the LCOCV statistics of all these ten scoring functions, we found that they all performed well in certain clusters (e.g. trypsin and *β*-secretase I) and poorly in some other clusters (e.g. HIV protease and factor Xa). The reasons for the large spread of performance across the different clusters are manifold, and a comprehensive analysis for each protein family would be beyond the scope of this study. As pointed out in [22], eliminating all the HIV protease complexes leads to an imbalance between the training and test sets because HIV protease inhibitors are on average much larger than the ligands of the other targets. This illustrates that the LCOCV results should not be directly interpreted as performance measures on particular protein families. Moreover, the limited size of many clusters and the small range of measured binding affinity values therein make a satisfactory prediction of the ranking rather challenging.

**Table 5:** **Leave-cluster-out cross validation results of the four models on the 23 protein families (A to W) and 3 multi-family (X to Z) clusters of PDBbind v2009 refined set (N = 1740) in terms of root mean square error RMSE, standard deviation SD in linear correlation, Pearson correlation coefficient Rp and Spearman correlation coefficient Rs**

					MLR::Cyscore				RF::Cyscore				RF::CyscoreVina				RF::CyscoreVinaElem	
Cluster name	Cluster	N	RMSE	SD	Rp	Rs	RMSE	SD	Rp	Rs	RMSE	SD	Rp	Rs	RMSE	SD	Rp	Rs
HIV protease	A	188	1.65	1.53	0.259	0.216	1.70	1.51	0.310	0.201	1.76	1.56	0.182	0.105	1.77	1.56	0.166	0.129
trypsin	B	74	1.24	1.11	0.612	0.695	1.10	1.11	0.610	0.636	0.96	0.97	0.723	0.700	0.93	0.93	0.751	0.715
carbonic anhydrase	C	57	2.47	1.35	0.473	0.343	2.44	1.43	0.368	0.264	2.60	1.37	0.448	0.372	2.33	1.35	0.481	0.234
thrombin	D	53	1.52	1.40	0.702	0.676	1.50	1.44	0.680	0.611	1.47	1.45	0.675	0.675	1.46	1.40	0.699	0.680
protein tyrosine phosphatase	E	32	1.23	1.06	0.411	0.313	1.30	1.10	0.338	0.268	1.36	0.98	0.538	0.542	1.23	0.89	0.643	0.615
factor Xa	F	32	1.18	0.96	0.604	0.634	1.54	1.13	0.367	0.356	1.53	1.02	0.533	0.498	1.61	1.07	0.470	0.470
urokinase	G	29	1.15	1.14	0.643	0.602	1.10	1.14	0.642	0.645	1.25	1.27	0.516	0.436	1.05	1.06	0.699	0.624
different similar transporters	H	29	0.96	0.96	0.285	0.122	1.27	0.99	0.056	-0.040	1.10	0.98	0.188	0.077	1.01	0.93	0.354	0.123
c-AMP dependent kinase	I	17	1.32	1.15	0.537	0.537	1.16	1.11	0.582	0.602	0.94	0.91	0.748	0.664	1.06	0.91	0.747	0.644
*β*-glucosidase	J	17	1.03	0.78	0.383	0.316	1.04	0.76	0.444	0.365	0.92	0.72	0.518	0.443	1.05	0.68	0.597	0.649
antibodies	K	16	1.41	1.43	0.693	0.706	1.67	1.76	0.455	0.466	1.47	1.51	0.645	0.643	1.36	1.33	0.739	0.777
casein kinase II	L	16	0.75	0.58	0.538	0.358	0.76	0.58	0.535	0.330	0.90	0.60	0.493	0.322	0.97	0.61	0.454	0.309
ribonuclease	M	15	1.12	1.20	0.230	0.340	1.07	1.06	0.505	0.281	1.11	0.99	0.595	0.481	1.23	1.03	0.551	0.493
thermolysin	N	14	1.15	1.14	0.680	0.635	0.98	1.03	0.748	0.648	1.04	1.12	0.696	0.565	0.97	1.05	0.738	0.636
CDK2 kinase	O	13	1.06	0.80	0.841	0.812	1.14	1.01	0.733	0.817	1.14	1.02	0.729	0.661	1.12	1.14	0.640	0.525
glutamate receptor 2	P	13	1.08	0.85	0.070	0.096	1.09	0.85	0.120	0.097	1.08	0.85	0.116	0.121	1.00	0.84	0.123	0.016
P38 kinase	Q	13	0.55	0.57	0.834	0.896	0.76	0.66	0.762	0.757	0.95	0.62	0.799	0.764	0.59	0.51	0.870	0.896
*β*-secretase I	R	12	1.44	1.33	0.892	0.725	1.57	1.51	0.858	0.620	1.54	1.51	0.860	0.687	1.43	1.31	0.895	0.687
tRNA-guanine transglycosylase	S	12	0.90	0.95	0.463	0.544	1.06	1.04	0.212	0.375	0.87	0.95	0.457	0.403	0.87	0.95	0.457	0.522
endothiapepsin	T	11	1.18	1.30	0.435	0.215	1.28	1.35	0.358	0.210	1.35	1.36	0.345	0.215	1.36	1.27	0.480	0.210
*α*-mannosidase 2	U	10	1.67	1.63	-0.004	0.248	1.65	1.62	0.116	0.188	1.73	1.62	0.089	0.176	1.83	1.63	0.053	0.103
carboxypeptidase A	V	10	2.13	1.99	0.479	0.523	1.90	1.89	0.556	0.370	1.82	1.76	0.632	0.467	1.77	1.54	0.734	0.685
penicillopepsin	W	10	1.71	1.87	0.339	0.188	1.78	1.94	0.236	0.188	1.81	1.96	0.183	0.030	1.91	1.99	0.078	-0.030
families with 4-9 complexes	X	386	1.73	1.71	0.500	0.577	1.61	1.60	0.587	0.598	1.58	1.56	0.610	0.612	1.54	1.53	0.630	0.632
families with 2-3 complexes	Y	340	1.64	1.64	0.510	0.495	1.64	1.63	0.522	0.505	1.55	1.55	0.583	0.580	1.51	1.52	0.608	0.595
singletons	Z	321	1.76	1.74	0.407	0.417	1.81	1.75	0.397	0.395	1.70	1.68	0.476	0.467	1.67	1.65	0.503	0.507
average			1.35	1.24	0.493	0.470	1.38	1.27	0.465	0.414	1.37	1.23	0.515	0.450	1.33	1.18	0.545	0.479
standard deviation			0.41	0.38	0.216	0.217	0.38	0.37	0.209	0.212	0.39	0.36	0.211	0.211	0.39	0.35	0.228	0.251

While results on standard cross validation might be too optimistic, results on leave-cluster-out cross validation might be too pessimistic. Here we want to emphasize that LCOCV is only suitable for estimating the performance of a generic scoring function on a truly new target protein that does not belong to a cluster represented by any of the proteins in the training set, but this constitutes a very uncommon scenario in real-life applications because it is rare for a target protein not to have high sequence similarity to any other protein in a diverse and large training set. In fact, such type of complexes should never be eliminated from a training set. Instead, the training set composition should reflect as closely as possible the actual complexes on which the scoring function is to be applied. Consequently, LCOCV is not appropriate to evaluate generic scoring functions, as previously argued [30].

### Machine-learning scoring functions are significantly more accurate than classical scoring functions with fixed functional forms

Table 6 compares Cyscore, RF::Cyscore, RF::CyscoreVina and RF::CyscoreVinaElem against 21 other scoring functions on PDBbind v2007 core set (N = 195), with RF::CyscoreVinaElem performing best in terms of Rp, Rs and SD. It is worth noting that the top four scoring functions are all trained with RF.

**Table 6:** **Prediction performance of 25 scoring functions evaluated on PDBbind v2007 core set (N = 195) in terms of Pearson correlation coefficient Rp, Spearman correlation coefficient Rs and standard deviation SD in linear correlation on the test set**

Scoring function	Rp	Rs	SD
RF::CyscoreVinaElem	0.803	0.798	1.42
RF-Score::Elem-v2	0.803	0.797	1.54
SFCscoreRF	0.779	0.788	1.56
RF-Score	0.774	0.762	1.59
ID-Score	0.753	0.779	1.63
RF::CyscoreVina	0.749	0.759	1.58
SVR-Score	0.726	0.739	1.70
RF::Cyscore	0.687	0.694	1.73
Cyscore	0.660	0.687	1.79
X-Score::HMScore	0.644	0.705	1.83
DrugScoreCSD	0.569	0.627	1.96
SYBYL::ChemScore	0.555	0.585	1.98
DS::PLP1	0.545	0.588	2.00
GOLD::ASP	0.534	0.577	2.02
SYBYL::G-Score	0.492	0.536	2.08
DS::LUDI3	0.487	0.478	2.09
DS::LigScore2	0.464	0.507	2.12
GlideScore-XP	0.457	0.435	2.14
DS::PMF	0.445	0.448	2.14
GOLD::ChemScore	0.441	0.452	2.15
SYBYL::D-Score	0.392	0.447	2.19
DS::Jain	0.316	0.346	2.24
GOLD::GoldScore	0.295	0.322	2.29
SYBYL::PMF-Score	0.268	0.273	2.29
SYBYL::F-Score	0.216	0.243	2.35

The scoring functions are sorted in the descending order of Rp. RF::CyscoreVinaElem and Cyscore rank 1st and 9th respectively in terms of Rp. The statistics for the other 21 scoring functions are collected from [8, 22, 31].

### Substituting RF for MLR and incorporating more features and training samples strongly improves Cyscore

**Figure 2:**
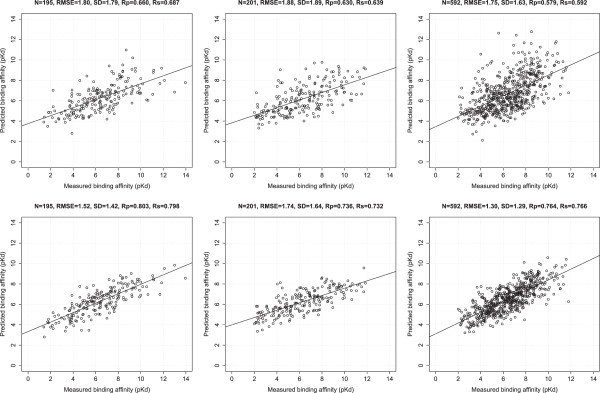
**Correlation plots of predicted binding affinities against measured ones.** Top row: Cyscore. Bottom row: RF::CyscoreVinaElem. Left column: PDBbind v2007 benchmark (N = 195), with RF::CyscoreVinaElem trained on 1105 complexes. Center column: PDBbind v2012 benchmark (N = 201), with RF::CyscoreVinaElem trained on 2696 complexes. Right column: PDBbind v2013 round-robin benchmark (N = 592), with RF::CyscoreVinaElem trained on 2367 complexes.

Figure 2 compares the prediction performance of Cyscore and RF::CyscoreVinaElem, with RF::CyscoreVinaElem improving Cyscore by -0.28 in RMSE, -0.37 in SD, +0.143 in Rp and +0.111 in Rs on the PDBbind v2007 benchmark, by -0.14 in RMSE, -0.25 in SD, +0.106 in Rp and +0.093 in Rs on the PDBbind v2012 benchmark, and by -0.40 in RMSE, -0.29 in SD, +0.187 in Rp and +0.184 in Rs on the PDBbind v2013 round-robin benchmark.

These results show that RF::CyscoreVinaElem performed consistently better than Cyscore on all the three benchmarks. It is important to note that, in each benchmark, both scoring functions used the same non-overlapping training and test sets. Taken together, these results show that one can develop a much more accurate scoring function out of an existing one simply by changing the regression model from MLR to RF and incorporating more structural features and training samples.

### Sensitivity analysis of the RF model can determine feature importance

**Figure 3:**
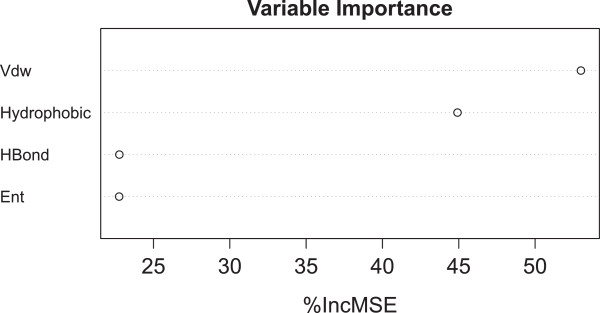
**RF::Cyscore feature importance estimated on internal OOB data of the 1105 complexes from PDBbind v2007 refined set.** The four features are hydrophobic free energy (Hydrophobic), van der Waals interaction energy (Vdw), hydrogen bond interaction energy (HBond) and ligand’s conformational entropy (Ent). The %IncMSE value of a particular feature was computed as the percentage of increase in mean square error observed in OOB prediction when that features was randomly permuted.

Unlike classical scoring functions, RF-based scoring functions can hardly be explicitly expressed as a mathematical equation like Eq. 1. Therefore it is useful to employ the variable importance tool of RF to estimate the importance of each feature by randomly permuting its training values, and the feature leading to the largest variation in the predicted binding affinity on the OOB data can be regarded as the most important for a particular training set. Figure 3 plots the percentage of increase in mean square error (%IncMSE) observed when each of the 4 Cyscore features used to train RF was noised up. All the 4 features turned out to be important (%IncMSE>20), with van der Waals interaction energy (Vdw) and hydrophobic free energy (Hydrophobic) being relatively more important (%IncMSE>40). Correctly estimating variable importance can assist in feature selection and in understanding ligand binding.

## Conclusions

In this study we have demonstrated that, on one hand, the multiple linear regression (MLR) model used in many scoring functions like Cyscore does not improve its performance in the presence of abundant training samples. This is a particularly significant drawback for MLR-based scoring functions because they cannot benefit from the future availability of more experimental data. On the other hand, RF-based scoring functions can comprehensively capture the non-linear nature in the data and thus assimilate data significantly better than MLR-based scoring functions. Most importantly, feeding more training samples to RF can increases its prediction performance. Under this circumstance, improvements with dataset size can only be gained with the appropriate regression model. Simply changing the regression model of Cyscore from MLR to RF and expanding the feature set and the sample set can significantly increase the prediction accuracy. The performance gap between MLR-based and RF-based scoring functions will be further widened by the future availability of more and more X-ray crystal structures.

Moreover, classical empirical scoring functions usually rely on complicated energetic contributions that must be carefully devised from intermolecular interactions, whereas RF-based scoring functions can also effectively exploit features as simple as occurrence count of intermolecular contacts. It has also been shown that functional group contributions in protein-ligand binding are non-additive. This means new features cannot be easily incorporated into an existing MLR model. In this study we have shown that using more structural features appropriately can also substantially enhance the prediction accuracy of RF, as can be seen in the comparison between RF::CyscoreVinaElem and RF::Cyscore. This further stresses the importance of substituting RF for MLR in scoring function development.

## Electronic supplementary material

Additional file 1: **CV.** This CSV file contains the PDB IDs and measured binding affinities of the protein-ligand complexes in the five partitions of PDBbind v2013 refined set for cross validation purpose. (CSV 35 KB)

Additional file 2: **Lcocv.** This CSV file contains the PDB IDs and measured binding affinities of the protein-ligand complexes in the 23 protein families and 3 multi-family clusters of PDBbind v2009 refined set for leave-cluster-out cross validation purpose. (CSV 20 KB)

Additional file 3: **Stat.** This Excel file contains the prediction performance of MLR::Cyscore, RF::Cyscore, RF::CyscoreVina and RF::CyscoreVinaElem trained with varying numbers of samples and tested on the PDBbind v2007, v2012 and v2013 benchmarks and also in the standard 5-fold and leave-cluster-out cross validations in terms of root mean square error RMSE, standard deviation SD in linear correlation on the test set, Pearson correlation coefficient Rp, Spearman correlation coefficient Rs and Kendall correlation coefficient Rk. (XLSX 28 KB)
